# Prevalence and Factors Associated with Behavioral Problems in 5-Year-Old Children Born with Cleft Lip and/or Palate from the Cleft Collective

**DOI:** 10.1177/10556656221119684

**Published:** 2022-09-09

**Authors:** Samantha Berman, Gemma C. Sharp, Sarah J. Lewis, Rachel Blakey, Amy Davies, Kerry Humphries, Yvonne Wren, Jonathan R. Sandy, Evie Stergiakouli

**Affiliations:** 1 Cleft Collective, 1980 University of Bristol, Bristol, UK; 2 Population Health Sciences, Bristol Medical School, Bristol, UK; 3 MRC Integrative Epidemiology Unit, 1980 University of Bristol, Bristol, UK; 4 Bristol Speech and Language Therapy Research Unit, North Bristol NHS Trust, Bristol, UK

**Keywords:** cleft lip and palate, cleft collective, strengths and difficulties questionnaire (SDQ), behavioral problems, behavioral difficulties, hyperactivity, emotional difficulties, sociodemographic factors, ALSPAC

## Abstract

**Objectives:**

To determine the UK prevalence of behavioral problems in 5-year-old children born with isolated or syndromic cleft lip and/or palate (CL/P) compared to the general population and identify potentially associated factors.

**Design:**

Observational study using questionnaire data from the Cleft Collective 5-Year-Old Cohort study and three general population samples.

**Main Outcome Measure:**

The Strengths and Difficulties Questionnaire (SDQ).

**Participants:**

Mothers of children (age: 4.9-6.8 years) born with CL/P (*n*  =  325). UK general population cohorts for SDQ scores were: Millennium Cohort Study (MCS) (*n*  =  12  511), Office of National Statistics (ONS) normative school-age SDQ data (*n*  =  5855), and Avon Longitudinal Study of Parents and Children (ALSPAC) (*n*  =  9386).

**Results:**

By maternal report, 14.2% of children born with CL/P were above clinical cut-off for behavioral problems, which was more likely than in general population samples: 7.5% of MCS (OR  =  2.05 [1.49-2.82], *P* < 0.001), 9.8% of ONS (OR  =  1.52 [1.10-2.09], *P*  =  0.008), and 6.6% of ALSPAC (OR  =  2.34 [1.70-3.24], *P* < 0.001). Children in the Cleft Collective had higher odds for hyperactivity, emotional and peer problems, and less prosocial behaviors. Maternal stress, lower maternal health-related quality of life and family functioning, receiving government income support, and maternal smoking showed evidence of association (OR range: 4.41-10.13) with behavioral problems, along with maternal relationship status, younger age, and lower education (OR range: 2.34-3.73).

**Conclusions:**

Findings suggest elevated levels of behavioral problems in children born with CL/P compared to the general population with several associated maternal factors similar to the general population.

## Introduction

Cleft lip and/or palate (CL/P) is one of the most common congenital anomalies affecting around 1000 babies born in the United Kingdom every year. Approximately 1 in 650 children are born with an orofacial cleft in the United Kingdom.^1^ While prognosis is generally good, children born with CL/P undergo several operations, may show physical signs of scarring and can experience breathing, feeding, speech, dental, and hearing problems.^2^

In recent years, research has focused on the wider implications of cleft, particularly the psychological and social impacts, in addition to cosmetic and functional impacts.^3–6^ Speech and language difficulties, poor self-image, and negative social experiences, compounded by the stress of ongoing medical treatment, are thought to increase the risk of behavioral problems in children born with CL/P.^7–9^

Psychological functioning and behavioral problems in children are strong predictors of future mental health.^10^ Studies have linked difficulties in childhood to academic underachievement, psychosomatic disorders, unemployment, and an overall reduced quality of life.^10–15^ As an early diagnosis and intervention services for mental health concerns are known to proffer better health outcomes and diminish economic and societal costs, there is an impetus to identify children who may be at an elevated risk for developing difficulties.^10^

Some children born with CL/P present with subclinical mental health and wellbeing needs^16^ which are negatively impacted when the cleft is combined with additional conditions and stresses.^5^ Evidence from longitudinal and cross-sectional studies suggests that children born with CL/P present with behavioral problems at ages 5 and 10 years more frequently than children without CL/P from the general population.^17,18^ However, large-scale individual studies on clinical populations are rare and more research is required to ascertain the prevalence of behavioral problems and to examine the possible unmet needs of this group.^6,19–21^ In addition, some studies did not report any evidence of increased behavioral difficulties in children born with cleft.^22^

There is evidence to suggest that children born with CL/P struggle with difficulties related to conduct,^23^ social skills,^23–26^ self-regulation,^27^ and hyperactivity.^18^ Such behavioral problems, along with cleft-related functional challenges and aesthetic differences, have been associated with teasing,^28^ stigma,^9,29^ social isolation,^12,25,30,31^ and psychological adjustment issues.^20,26^

Sociodemographic factors and maternal characteristics are known to affect mental health and behavioral outcomes in children.^32^ Studies based on large cohorts, such as the Avon Longitudinal Study of Parents and Children (ALSPAC) and the Millennium Cohort Study (MCS), have contributed to understanding of the relative predictive power of parental health and sociodemographic variables on childhood behavioral problems in the general UK population.^33,34^ For example, financial difficulties, housing tenure, younger maternal age at birth, and marital status have been observationally associated with attention deficit/hyperactivity disorder (ADHD) in 7-year old children from the general population.^35^ These associations have not been well explored in a cleft population.

The aim of this study was to estimate the prevalence of behavioral problems in 5-year-old children born with CL/P and compare it to that estimated in children of similar age from the general population. In addition, maternal, familial, and cleft-related factors were tested for association with behavioral problems in children born with CL/P.

## Methods

### Study Sample

The current study used cross-sectional baseline questionnaire data from the Cleft Collective 5-Year-Old Cohort from birth years 2008 to 2014. The Cleft Collective is an ongoing UK-wide longitudinal study, comprising a Birth Cohort and a 5-Year-Old Cohort, established to investigate the causes, best treatments, and the psychological impact of cleft on affected individuals and their families. Data from the Cleft Collective are available for clinical and academic communities to access and use to address a range of cleft-related research questions. More information on the study and how to access the dataset is available at http://www.bristol.ac.uk/cleft-collective/professionals/access/. A detailed description of recruitment procedures and the study's development can be found elsewhere.^36^

### Strengths and Difficulties Questionnaire

The Strengths and Difficulties Questionnaire (SDQ) was given to all parents/guardians in the Cleft Collective 5-year old cohort as a part of a larger baseline questionnaire. The SDQ is one of the most widely used screening tools, adopted globally as a measure of mental health.^37,38^ The 25-item survey assesses a child's behavioral problems across five subscales: emotional symptoms, conduct problems, hyperactivity, peer-related behavioral problems, and prosocial behavior.^39^ The instruments’ validity and reliability, including internal consistency, test–retest reliability, and inter-rater agreement have been established, and it has been shown to be strongly correlated with other tools.^39–41^ Previous studies have shown that although SDQ subscales vary, parent-reported total difficulties scores have good internal reliability.^42^

In this study, parents completed the SDQ on behalf of the study child. Parents answered questions relating to their child's behavior using a three-point rating scale: not true (0), somewhat true (1), and certainly true (2). Each subscale, comprised of five questions, was then scored on a scale of 0-10. Anchor points were reversed for the four scales measuring difficulties (emotional, conduct, hyperactivity, and peer problems) so that high scores in these categories represent greater difficulties. Total difficulties scores were calculated by summing scores from four subscales: conduct problems, hyperactivity, emotional, and peer problems. The prosocial scale was scored as is, with high scores in this domain indicating fewer difficulties. For the purposes of the current study, reports from mothers were only used (ie, not partners or other guardians) for consistency and because these were more complete.

Given a skewed distribution of the total difficulties score and each of the subscale scores, SDQ scores were dichotomized based on validated cut-points. Scores above subscale-specific thresholds (≥4 for conduct, ≥5 for emotional, ≥7 for hyperactivity, ≥4 for peer problems, and ≤6 for prosocial) were designated as “cases,” signaling being at high risk of these behavioral problems and scores below were classified as “non-cases.”^39,40^ The overall total difficulties score ≥17 is henceforth used to indicate an individual at high risk of behavioral problems.

### Maternal, Familial, and Cleft-Related Factors Tested for Association with Behavioral Problems

From the Cleft Collective 5-year old cohort baseline questionnaire completed by the mothers, the following variables were generated and tested for association with behavioral problems: child's sex assigned at birth (male, female; obtained from parental report and medical notes, where this information was not available, sex was assumed by the Cleft Collective operations team based on name and confirmed with cleft teams); cleft type (cleft lip, cleft palate, cleft lip, and palate; derived from multiple sources including parental report and medical notes); cleft associated with a syndrome (no, yes confirmed, or suspected; derived from multiple sources including parental report and medical notes); maternal age at conception (dichotomized as >24 years, ≤24 years; self-reported); mother's ethnicity (Asian, Black, other minority ethnic group, white; self-reported); mother's highest educational qualification (no university degree, university degree, or equivalent; self-reported); derived household annual income [<£20,000, ≥£20,000; self-reported; mothers and partners were asked about their annual income separately; the threshold of £20,000 was used as an indicator of “Living Wage” (ie, the minimum income necessary for a person to meet their basic living needs such as food and shelter in the UK. If they lived together, incomes were summed together. If mother lived on their own, only mother's income was reported); mother receives government income support (no, yes; self-reported); number of biological children prior to the study child (parity) (0, ≥1; self-reported); mother currently smokes cigarettes (no, yes; self-reported); mother's marital status (married/partnered, single/divorced/widowed; self-reported); mother currently drinks alcohol (no, yes; self-reported).

The Perceived Stress Scale (PSS) is a measure of quantifying a person's personal stress. Respondents were asked to indicate how often they may have reacted to certain events in a particular way within the last month using a 5-point Likert scale (0 = “Never” to 4 = “Very Often”). Within the Cleft Collective, mothers scored a mean of 14.91 with a standard deviation of 7.27 (median  =  15; IQR  =  10-20). The PSS scores were dichotomized on the 50^th^ percentile (%) mark for the study cohort.

The Pediatric Quality of Life (PedsQL) Family Impact Module (FIM)^43^ is a measure quantifying the impact of a pediatric chronic health condition on the family. Parents were asked to complete a 36-item questionnaire, which included questions on the physical, emotional, social, and cognitive functioning, communication, worry, daily activities, and family relationships. A total score was calculated with higher values indicating better functioning (values range from 0 to 100). Summary scores were also calculated, and they included the family functioning summary score and the Maternal Health Related Quality of Life (HRQoL) summary score. The family functioning summary score was calculated using 8 items from the daily activities and family relationship scales. The HRQoL summary score was calculated using 20 items from the Physical, Emotional, Social, and Cognitive Functioning scales. Within our sample mothers scored a mean of 82.72 with a standard deviation of 19.06 (median  =  89.58; IQR  =  70.83-99.31) for the total FIM score; a mean of 87.63 with a standard deviation of 19.65 (median  =  100; IQR  =  75-100) for the family functioning summary score; and a mean of 80.62 with a standard deviation of 20.33 (median  =  86.88; IQR  =  65.63-100) for the total FIM score. The PedsQL FIM, PedsQL Family Functioning, and PedsQL Maternal HRQoL summary scores were dichotomized on the 50th percentile (%) mark for the study cohort.

### General Population Estimates (Control Samples)

Published estimates from three large cohorts were used to approximate general population SDQ scores in the UK. SDQ scores differ by age, sex, and context-specific factors, necessitating careful interpretations of normative data averages used to make cross-cohort comparisons.^37,44^ The MCS was used as the primary control group in this study, as the age of participants and recruitment timeline most closely match the Cleft Collective.^45^ The MCS third survey sweep provided SDQ estimates from a UK-wide sample of 5-year-olds (*n*  =  12 511),^45^ and estimates are published https://cls.ucl.ac.uk/cls-studies/millennium-cohort-study/mcs-age-5-sweep/. The study included children born between September 2000 and January 2002 and intentionally over-sampled children of ethnic minority backgrounds as well as those living in resource poor areas, who may otherwise have lower response rates, in order to ensure a representative sample.^45^ The MCS aligned with the Cleft Collective in terms of age, timing, and geographical representation.

In addition, normative (Norms) school-age SDQ data from the UK's Office of National Statistics (ONS), split by sex- and age-band are published^46^ and are widely cited in the literature.^18,47^ Estimates were established from a large national survey carried out by the ONS in 1999 and included a representative sample of (*n*  =  5855) parent-reported SDQ data for children aged 5 to 10 years. More information about the study sample can be found elsewhere.^47^ These general population estimates represent the most widely used British “norms,” but their comparability with the Cleft Collective is limited by large age-bands and temporal differences.

Finally, mean SDQ scores were calculated in the ALSPAC study, which is a transgenerational prospective study, which recruited pregnant women resident in Avon, UK with delivery dates between 1 April 1991 and 31 December 1992.^48,49^ Mothers (*n*  =  9386) from the ALSPAC study completed the SDQ on their children between the ages of 3.7 and 5.4 years (mean  =  4.0 SD  =  0.12). This cohort provided general population estimates from a large sample and offered sex-specific estimates. Though there was a slight age difference between the samples, the age bracket for this control was small and overlapped the study cohort. The comparability of this sample with the Cleft Collective was limited by generational and geographical differences. The study website contains details of all the data that are available through a fully searchable data dictionary (http://www.bristol.ac.uk/alspac/researchers/our-data/) and variable search tool. More information can be found online (http://www.bristol.ac.uk/alspac/researchers/participants/).

Ethical approval for the ALSPAC study was obtained from the ALSPAC Law and Ethics committee and local research ethics committees. Published data on SDQ from the Millennium Birth Cohort and the UK norms were used so separate ethics approval was not sought for these studies.

### Statistical Analysis

SDQ scores were calculated for all participants in Stata, using the scoring guide published.^46^ Stata was used to calculate mean scores and standard deviations (SD) for each SDQ subscale.

Odds ratios of children in the Cleft Collective having behavioral problems compared to those in the general population cohorts were calculated. Odds ratios and 95% confidence intervals (CIs) were calculated to compare the proportion of high total difficulties score “cases” in the Cleft Collective to those in each of the three cohorts. Analyses were repeated for “cases” across each SDQ subscale. All analyses were repeated stratified by sex.

Logistic regression was used to assess associations between dichotomized SDQ scores (dependent variable) and maternal, familial, and cleft-related (independent) variables. In each analysis, binary SDQ scores defined the dependent variable and the maternal factors were used as binary independent variables, except cleft type that was treated as a nominal categorical variable. Odds ratios (ORs) with 95% CIs and sex-adjusted ORs for each of the variables across subscales were reported.

## Results

### Sample Description

Eight hundred and ninety-eight mothers participated in the Cleft Collective 5-Year-Old Cohort at the point of analysis. Data to calculate SDQ total difficulties scores were available for 325 5-year-olds with CL/P at recruitment resulting in a response rate of 36.2%. Data on the prosocial subscale were only available on 324 5-year-old children (the prosocial scale is not required to calculate the SDQ total difficulties scores). Table 1 summarizes the maternal and sociodemographic characteristics of the Cleft Collective study sample, along with the number and percentage of behavioral difficulties by variable.

**Table 1.** Overview of Sociodemographic, Maternal Characteristics and Childhood Behavioral Problems across the Cleft Collective for Mothers Who Returned the Baseline Questionnaire at Age 5.

Sociodemographic and Maternal Characteristics	*n*(%) Total		*n* SDQ Total Difficulties Score ≥17 *n*(%) Cases	
**SDQ total difficulties scores calculated (*n* = 325)**				
Yes	325	(100.0%)	46	(100.0%)
**Sex (*n* = 320)**				
Male	166	(51.9%)	30	(65.2%)
Female	154	(48.1%)	16	(34.8%)
**Cleft type (*n* = 163)**				
Cleft lip	40	(24.5%)	6	(24.0%)
Cleft palate	56	(34.4%)	8	(32.0%)
Cleft lip and palate	67	(41.1%)	11	(44.0%)
**Syndrome (*n* = 90)**				
No	80	(88.9%)	10	
Yes	10	(11.1%)	<5^a^	
**Maternal age at conception (*n* = 310)**				
>24 years	259	(83.5%)	26	(63.4%)
≤24 years	51	(16.5%)	15	(36.6%)
**Ethnicity (*n* = 314)**				
White	292	(93.0%)	42	
Black, Asian, Minority Ethnic	22	(7.0%)	<5^a^	
**Maternal education (*n* = 305)**				
No university degree	145	(47.5%)	13	(30.2%)
University degree or equivalent	160	(52.5%)	30	(69.8%)
**Household income (*n* = 299)**				
<£20,000 annually	214	(71.6%)	29	(69.0%)
≥£20,000 annually	85	(28.4%)	13	(31.0%)
**Mother receives income support (*n* = 309)**				
No	290	(93.9%)	36	(80.0%)
Yes	19	(6.1%)	9	(20.0%)
**Parity (*n* = 314)**				
0	169	(53.8%)	27	(62.8%)
≥1	145	(46.2%)	16	(37.2%)
**Mother smokes (*n* = 146)**				
No	105	(71.9%)	10	(43.5%)
Yes	41	(28.1%)	13	(56.5%)
**Marital status (*n* = 319)**				
Married/partnered	276	(86.5%)	33	(71.7%)
Single/divorced/widowed	43	(13.5%)	13	(28.3%)
**Mother consumes alcohol (*n* = 323)**				
No	102	(31.6%)	14	(31.1%)
Yes	221	(68.4%)	31	(68.9%)
**Maternal PSS (*n* = 306)** ^b^				
<50%	153	(50.0%)	<5^a^	
≥50%	153	(50.0%)	39	
**Total PedsQL FIM (*n* = 320)** ^b^				
<50%	162	(50.6%)	9	(19.6%)
≥50%	158	(49.4%)	37	(80.4%)
**PedsQL (family functioning) (*n* = 321)** ^b^				
<50%	190	(59.2%)	13	(28.3%)
≥50%	131	(40.8%)	33	(71.7%)
**PedsQL (maternal HRQoL) (*n* = 279)** ^b^				
<50%	142	(50.9%)	6	(15.0%)
≥50%	137	(49.1%)	34	(85.0%)

Abbreviations: HRQOL, health-related quality of life; PEDSQL FIM, Pediatric Quality of Life Inventory Family Impact Module; PSS, Perceived Stress Scale; SDQ, Strengths and Difficulties Questionnaire.

^a^ Cell counts of less than 5 cannot be published for disclosure purposes, and % cannot be provided for the same reason.

^b^ Scores for these variables were dichotomized on the 50^th^ percentile (%) mark for the study cohort.

### Prevalence of Behavioral Problems Among the Cleft Collective 5-Year-Old Cohort

Overall, 14.2% of 325 children (age: 4.9-6.8 years) from the Cleft Collective study were at high risk of behavioral problems. Table 2 shows the frequency (*n*) and percentage (%) of “cases” in the Cleft Collective by SDQ subscale. In total, 18.1% of males and 10.4% of females in the cohort were classed as cases (data on SDQ and sex were available for 320 children) (Table 3). The hyperactivity and prosocial subscales showed the greatest sex disparities (hyperactivity: 22.9% of males and 14.3% of females, less prosocial behaviors: 22.4% of males and 15.6% of females) (Table 3).

**Table 2.** Prevalence of Behavioral Problems across SDQ Subscales in Children Born with CL/P from the Cleft Collective (Across the Total Sample).

**SDQ Subscale-Specific Thresholds for Cases**	***N* (%) Cases**	***N* (%) Non-cases**	**Total**
**Total difficulties ≥17**	46 (14.2%)	279 (85.8%)	325
**Conduct ≥4**	39 (12.0%)	286 (88.0%)	325
**Emotional ≥5**	43 (13.2%)	282 (86.8%)	325
**Hyperactivity ≥7**	60 (18.5%)	265 (81.5%)	325
**Peer problems ≥4**	42 (12.9%)	283 (87.1%)	325
**Prosocial ≤6^a^**	62 (19.1%)	262 (80.9%)	324

Abbreviation: SDQ, Strengths and Difficulties Questionnaire.

^a^ Total difficulties scores were calculated by summing scores from four subscales: conduct problems, hyperactivity, and emotional and peer problems. Data on the prosocial subscale were only available on 324 5-year-old children (the prosocial scale is not required to calculate the SDQ total difficulties scores). Lower scores in the prosocial scale (≤6) indicate behavioral problems.

**Table 3.** Prevalence of Behavioral Problems across SDQ Subscales in Children Born with CL/P from the Cleft Collective (Stratified by sex).

**SDQ Subscale-Specific Thresholds for Cases^a^**	***N* (%) Males**			***N* (%) Female**		
	***N* (%) Cases**	***N* (%) Non-cases**	**Total**	***N* (%) Cases**	***N* (%) Non-cases**	**Total**
**Total difficulties ≥17**	30 (18.1%)	136 (81. 9%)	166	16 (10.4%)	138 (89.6%)	154
**Conduct ≥4**	25 (15.1%)	141 (84.9%)	166	14 (9.1%)	140 (90.9%)	154
**Emotional ≥5**	23 (13.9%)	143 (86.1%)	166	19 (12.3%)	135 (87.7%)	154
**Hyperactivity ≥7**	38 (22.9%)	128 (77.1%)	166	22 (14.3%)	132 (85.7%)	154
**Peer problems ≥4**	27 (16.3%)	139 (83.7%)	166	15 (9.7%)	139 (90.3%)	154
**Prosocial ≤6^b^**	37 (22.4%)	128 (77.6%)	165	24 (15.6%)	130 (84.4%)	154

Abbreviation: SDQ, Strengths and Difficulties Questionnaire.

^a^ Data on SDQ and sex were available for 320 children.

^b^ Total difficulties scores were calculated by summing scores from four subscales: conduct problems, hyperactivity, and emotional and peer problems. Data on the prosocial subscale were only available on 324 5-year-old children (the prosocial scale is not required to calculate the SDQ total difficulties scores). Lower scores in the prosocial scale (≤6) indicate behavioral problems.

### Behavioral Problems in Cleft Compared to the General Population

Table 4 shows mean SDQ scores with SD and the frequency (*n*) and percentage (%) of cases and controls in the Cleft Collective and the three general population groups. Overall, the percentage of cases in the Cleft Collective (14.2%) was greater than the percentage within all three general population groups for total difficulties (MCS, 7.5%; ONS, 9.8%; ALSPAC, 6.6%).

**Table 4.** Summary of SDQ Scores and Behavioral Problems in the Cleft Collective 5-Year-Old Cohort Compared to General Population Estimates.

**SDQ Subscale-Specific Thresholds for Cases**	**Cohort**	***N* Total**	**Mean (SD)**	***N* (%) Cases**	***N* (%) Non-cases**
**Total difficulties ≥17**	Cleft Collective	325	9.03 (6.44)	46 (14.2%)	279 (85.8%)
	MCS	12 703	7.46 (5.44)	947 (7.5%)	11 756 (92.5%)
	ONS norms	10 298	8.60 (5.70)	1009 (9.8%)	9289 (90.2%)
	ALSPAC	9342	8.89 (4.56)	614 (6.6%)	8728 (93.4%)
**Conduct ≥4**	Cleft Collective	325	1.66 (1.71)	39 (12.0%)	286 (88.0%)
	MCS	12 808	1.38 (1.54)	1265 (9.9%)	11 543 (90.1%)
	ONS norms	10 298	1.60 (1.70)	1301 (12.6%)	8997(87.4%)
	ALSPAC	9371	1.96 (1.40)	1244 (13.0%)	8127 (87.0%)
**Emotional ≥5**	Cleft Collective	325	1.92 (2.13)	43 (13.2%)	282 (86.8%)
	MCS	12 780	1.53 (1.76)	950 (7.4%)	11 830 (92.6%)
	ONS norms	10 298	1.90 (2.00)	1166 (11.3%)	9132 (88.7%)
	ALSPAC	9385	1.44 (1.51)	430 (4.6%)	8955 (95.4%)
**Hyperactivity ≥7**	Cleft Collective	325	4.02 (2.79)	60 (18.5%)	265 (81.5%)
	MCS	12 760	3.37 (2.52)	1634 (12.8%)	11 126 (87.2%)
	ONS norms	10 298	3.60 (2.70)	1058 (14.6%)	8790 (85.4%)
	ALSPAC	9377	3.39 (2.37)	1342 (14.3%)	8035 (85.7%)
**Peer problems ≥4**	Cleft Collective	325	1.42 (1.79)	42 (12.9%)	283 (87.1%)
	MCS	12 786	1.22 (1.55)	1161 (9.1%)	11 625 (90.9%)
	ONS norms	1215	1.40 (1.70)	1215 (11.8%)	9083 (88.2%)
	ALSPAC	9384	1.52 (1.48)	1012 (10.8%)	8372 (89.2)
**Prosocial ≤6^a^**	Cleft Collective	324	8.33 (1.83)	62 (19.1%)	262 (80.9%)
	MCS	12 811	8.59 (1.64)	1610 (12.6%)	11 201 (87.4%)
	ONS norms	10 298	8.60 (1.60)	1094 (10.6%)	9204 (89.4%)
	ALSPAC	9372	7.04 (1.97)	2207 (23.5%)	7165 (76.5%)

Abbreviations: ALSPAC, Avon Longitudinal Study of Parents and Children; MCS, Millennium Cohort Study; ONS, Office of National Statistics; SD, standard deviation; SDQ, Strengths and Difficulties Questionnaire.

^a^ Total difficulties scores were calculated by summing scores from four subscales: conduct problems, hyperactivity, emotional and peer problems. Data on the prosocial subscale were only available on 324 5-year-old children (the prosocial scale is not required to calculate the SDQ total difficulties scores). Lower scores in the prosocial scale (≤6) indicate behavioral problems.

ORs, 95% CIs, and *P* values comparing the relative odds of cases in the Cleft Collective to the three general population estimates are reported in Table 5. There was strong evidence to suggest that 5-year-olds in the Cleft Collective were more likely to experience behavioral problems than those in all three general population cohorts: MCS (OR  =  2.05 [95% CI =  1.49-2.82]; *P* < 0.001), ONS Norms (OR  =  1.52 [95% CI  =  1.10-2.09]; *P*  =  0.008), and ALSPAC (OR  =  2.34 [95% CI  =  1.70-3.24]; *P* < 0.001).

**Table 5.** Odds Ratios for Behavioral Problems in the Cleft Collective Compared to General Population Samples.

SDQ Subscale-Specific Thresholds for Cases	Cohort	Odds Ratio	95% CIs	*P* Value
**Total difficulties ≥17**	Cleft Collective	1.00		
	MCS	2.05	1.49, 2.82	<0.001
	ONS norms	1.52	1.10, 2.09	0.008
	ALSPAC	2.34	1.70, 3.24	<0.001
**Conduct ≥4**	Cleft Collective	1.00		
	MCS	1.24	0.89, 1.75	0.207
	ONS norms	0.94	0.67, 1.32	0.735
	ALSPAC	0.89	0.63, 1.25	0.505
**Emotional ≥5**	Cleft Collective	1.00		
	MCS	1.90	1.37, 2.63	<0.001
	ONS norms	1.19	0.86, 1.66	0.268
	ALSPAC	3.18	2.27, 4.44	<0.001
**Hyperactivity ≥7**	Cleft Collective	1.00		
	MCS	1.54	1.16, 2.05	0.002
	ONS norms	1.88	1.41, 2.51	<0.001
	ALSPAC	1.36	1.02, 1.80	0.024
**Peer problems ≥4**	Cleft Collective	1.00		
	MCS	1.49	1.07, 2.07	0.011
	ONS norms	1.11	0.80, 1.54	0.432
	ALSPAC	1.23	0.88, 1.71	0.164
**Prosocial ≤6**	Cleft Collective	1.00		
	MCS	1.65	1.24, 2.18	<0.001
	ONS norms	1.99	1.50, 2.64	<0.001
	ALSPAC	0.77	0.58, 1.02	0.128

Abbreviations: ALSPAC, Avon Longitudinal Study of Parents and Children; CI, confidence interval; MCS, Millennium Cohort Study; ONS, Office of National Statistics; SDQ, Strengths and Difficulties Questionnaire.

Specifically, hyperactivity, emotional, and peer problems were more prevalent in the Cleft Collective than in all three general population estimates and less prosocial behaviors more prevalent than in two of the three cohorts (Table 4). For example, individuals in the cleft group were almost two times more likely to experience hyperactivity compared with individuals in the ONS norms group (OR 1.88 [95%CI =  1.41, 2.51]; *P* < 0.001) (Table 5). Furthermore, there was strong evidence suggesting greater odds of emotional problems among children in the Cleft Collective compared with MCS (OR  =  1.90 [95% CI  =  1.37-2.63]; *P* < 0.001) and ALSPAC (OR  =  3.18 [95% CI  =  2.27-4.44]; *P* < 0.001) (Table 5).

Sex-stratified analyses indicated that emotional problems were particularly prevalent among males (Supplementary Material 1), with males in the Cleft Collective nearly twice as likely as those in the MCS (OR  =  1.93 [95% CI  =  1.23-3.03]; *P*  =  0.004) and three times those in ALSPAC (OR  =  3.34 [95% CI  =  2.11-5.29]; *P* < 0.001) to experience emotional difficulties (Supplement 2). Sex-stratified analyses are reported in Supplementary Materials 1 and 2.

### Maternal, Familial, and Cleft-Related Factors Associated with Behavioral Problems in the Cleft Collective

Among mothers who completed the baseline questionnaire, response rates on questions informing predictors were high overall (>90%), with the exceptions of cleft type (*n*  =  163), smoking status (*n*  =  146), and syndrome (*n*  =  90) (Table 1).

**Figure 1.**
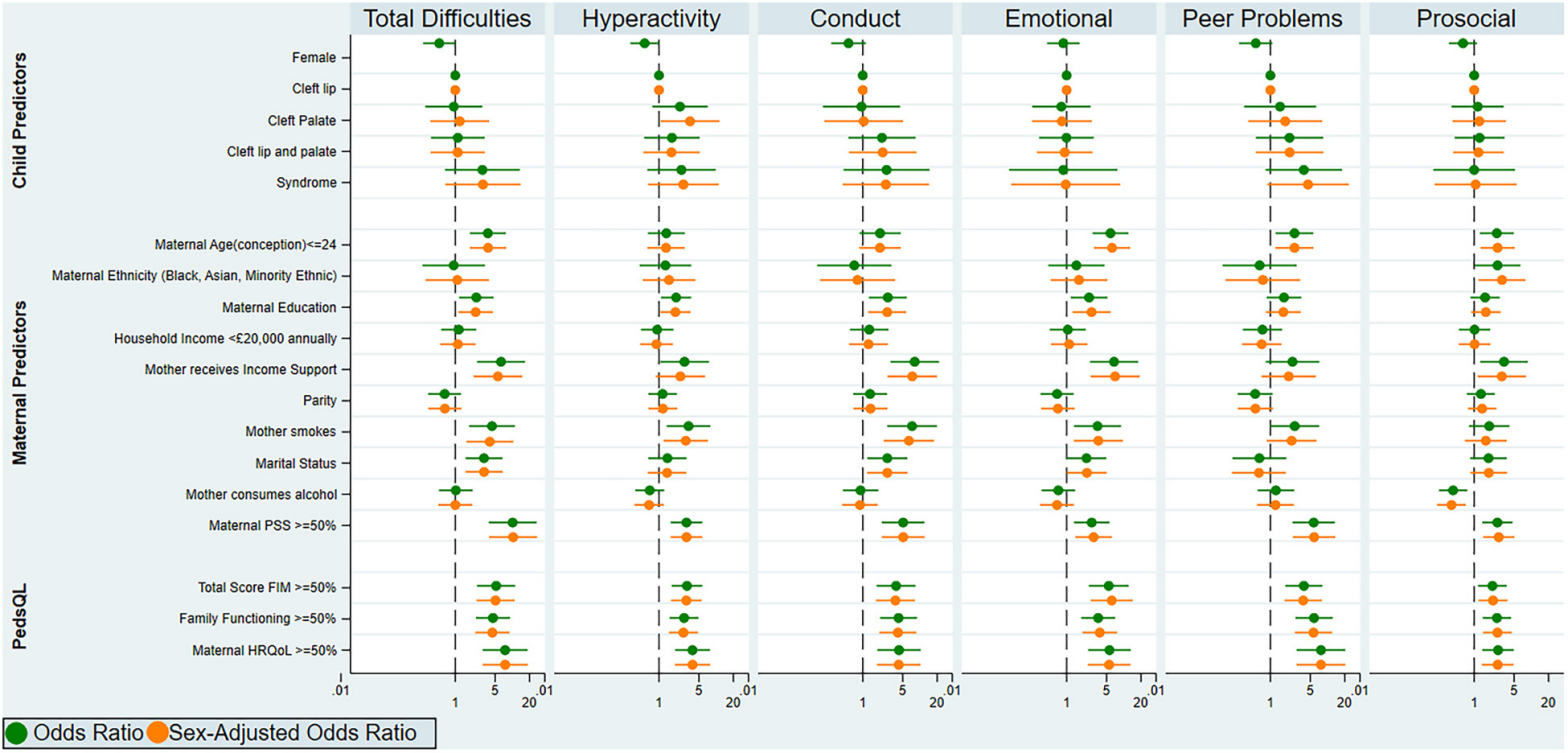
Associations between sociodemographic and maternal characteristics and behavioral problems among 5-year-olds with CL/P. This figure shows the results of logistic regression analyses between 13 predictor variables and case status; this binary classification system was established based on a population-based UK survey, (sdqinfo.com); odds ratios and sex-adjusted odds ratios with 95% confidence intervals are reported across SDQ subscales. Abbreviations: FIM, Family Impact Module; HRQoL, health-related quality of life; PedsQL; Pediatric Quality of Life Inventory; PSS, Perceived Stress Scale; SDQ, Strengths and Difficulties Questionnaire.

Figure 1 and Supplementary Materials 3 and 4 illustrate associations between maternal and familial variables and the odds of behavioral problems in the Cleft Collective, reporting ORs, sex-adjusted ORs, and 95% CI from a series of logistic regression analyses.

Higher maternal psychological stress, as indicated by scores above the 50^th^ percentile on the PSS, showed the strongest evidence of association, with higher odds of behavioral difficulties in children (OR = 10.13 [95% CI  =  3.87-26.51]; *P* < 0.001). Higher maternal and family psychological impact, as indicated by scores above the 50^th^ percentile on the PedsQL FIM,^50^ also showed strong evidence of association with higher odds of behavioral difficulties in children (OR = 5.20 [95% CI  =  2.42-11.19]; *P* < 0.001). The odds of behavioral problems in children whose families received government income support was 6-fold that of those whose families did not (OR = 6.35 [95% CI  =  2.42-16.68]; *P* < 0.001). However, there was little evidence of association of household income (< = £20 000 versus >£20 000 annually) with the odds of behavioral problems (OR = 1.15 [95% CI  =  0.57-2.34]; *P*  =  0.696) (Figure 1, Supplementary Material 3).

There was strong evidence of an association between the mother not being married (OR = 3.19 [95% CI  =  1.51-6.72]; *P*  =  0.002), being a smoker (OR = 4.41 [95% CI  =  1.75-11.13]; *P*  =  0.002), and younger maternal age at conception ≤24 years (OR = 3.73 [95% CI  =  1.81-7.72]; *P* < 0.001) with higher odds of behavioral difficulties in children born with CL/P. There was also good evidence of an association between lower maternal education (mother not having a higher education degree) (OR = 2.34 [95% CI  =  1.17-4.69]; *P*  =  0.016) and higher odds of behavioral problems in the study cohort (Figure 1, Supplementary Material 3).

Maternal ethnicity did not show evidence of association with the odds of behavioral problems in children, as defined by total difficulties score and across subscales. However, there was evidence of higher odds of less prosocial behaviors among children whose mothers self-reported their ethnicity as Asian, Black, or of another minority ethnic group in the UK compared to those whose mothers self-reported their ethnicity as white (Prosocial OR = 2.57 [95% CI  =  1.02-6.43]; *P*  =  0.044) (Supplementary Material 4).

## Discussion

In this study, an estimated 14.2% of 5-year-old children with CL/P experienced behavioral problems as measured by the SDQ total difficulties score. Findings suggested that children born with a cleft experience more behavioral problems and specifically more hyperactivity- and emotional-related difficulties compared to the general population. Greater peer problems and decreased prosocial behaviors were also more prevalent in the Cleft Collective relative to the general population, suggesting unmet needs in these areas.

Findings from this study also showed that known maternal and familial factors, which are associated with increased behavioral problems in the general population, were also associated with behavioral problems in children born with CL/P. Mainly, lower maternal education, lower income, lower maternal well-being, income, lower family functioning, and marital status showed associations with increased behavioral problems within the cohort.

### Comparison with Other Studies

These findings support previous research findings on the prevalence of behavioral problems in children born with cleft. Specifically, they are in agreement with previous investigations from the Cleft Care UK study, which reported increased hyperactivity-related difficulties (measured by parent-reported SDQ) in 5-year old children born with unilateral cleft lip and palate.^18^ Increased hyperactivity-related difficulties, but not other behavioral problems, were found in 6- to 12-year-old children born with cleft from the United States.^51^ In addition, a recent longitudinal study reported increased behavioral difficulties, as measured by the SDQ at ages 5 and 10 years, with boys born with cleft experiencing more difficulties.^17^ Emotional difficulties and reduced social competencies have also been found in school-aged children born with cleft.^18,25^ However, increased behavioral difficulties in children born with cleft were not reported in other studies, including a cross-sectional study of 11- to 16-year-old children born with cleft.^22^

Maternal and familial factors, including maternal smoking, mother not being married, younger maternal age at conception, lower maternal education, receiving government income support, higher maternal psychological stress, and higher maternal and family psychological impact, showed the strongest associations with behavioral problems in 5-year-olds born with CL/P. Maternal ethnicity did not appear to be associated with behavioral problems (as defined by total difficulties), and across subscales, however, there was evidence that maternal ethnicity was associated with less prosocial behaviors. For consistency with MCS findings^33^ and due to small sample sizes in all ethnic groups except white, ethnicity was explored as a binary variable and mothers in the cohort who identify as Asian, Black or any other minority ethnic group in the UK were grouped. We recognize that this is a major limitation of the exploration of ethnicity and that it makes it impossible to draw conclusions about the unique needs and experiences of individuals from different ethnic groups.

One of the strongest associations reported was between higher maternal PSS scores and behavioral problems at age 5. Previous studies have shown evidence of strong associations between maternal stress and child behavioral problems in both the general population and children born with cleft.^2,15,27,32,52^ Whether this association is stronger in children born with cleft warrants further investigation.^15^ In addition, it is not possible to establish causality and direction of effects as explained in the limitations section.

Family functioning has been linked to child psychological health and is known to be a particularly strong determinant of well-being in children born with chronic medical conditions.^53,54^ Findings from this study provided evidence of this association in children born with cleft, demonstrating the strong association between behavioral problems and measures of family functioning. Though the merits of the PedsQL FIM for measuring the impact of pediatric chronic health conditions have been well-established,^43,50,54,55^ the tool is relatively new to the cleft literature and this study joins few others.^52^ In one recent study using the PedsQL FIM, total scores and family functioning summary scores were reported to be higher following a diagnosis of CL/P compared to normative data.^52^ These results reinforce the utility of this tool in children born with cleft.^56^

### Strengths and Limitations

There are many key strengths to this study. Firstly, the sample size is large, geographically diverse across the UK and age specific. Secondly, UK cleft research is supported by standard provision and quality of patient care, relative to other countries. This homogeneity, augmented by the centralization of cleft care over the last two decades, offers major benefits for comparing patient outcomes and for evaluating the effectiveness of interventions implemented.^57^ Thirdly, three UK population cohorts of similar age to Cleft Collective were used as controls. Different biases and confounding structures might have been present in each cohort depending on recruitment and data collection procedures and the fact that results were in the same direction across all cohorts strengthens confidence in them. However, we note that data on the UK population cohorts have been collected up to 25 years earlier than the Cleft Collective.

The limitations of this study are inherent to its observational nature. Aside from true causal effects of cleft and maternal and familial variables on behavioral difficulties, the reported associations could be explained by bias or confounding variables. For example, reverse causation could be at play in the association between maternal perceived stress and behavioral problems in cleft. Having a child with behavioral problems could be causing increased psychological stress in the parents and not the other way around. Another possibility is that mothers with higher levels of stress could be reporting more behavioral problems in their children than mothers with lower levels of stress. The same could be true for family functioning. Other associations could be confounded by several measured and unmeasured factors. Alternative research designs are needed to infer causation, and Mendelian randomization offers one approach by reducing bias from confounding and reverse causation.^58^

Although more behavioral problems were observed among children with syndromic cleft versus those with non-syndromic cleft, only a third of the sample reported on syndromic status resulting in small sample sizes (*n* < 10) and making it impossible to statistically compare children with syndromic cleft versus those with non-syndromic cleft.

In this study, clinical diagnoses of conditions, such as ADHD, autism, dyslexia, specific language impairment, and developmental delay, which could be overrepresented in cleft populations, were not investigated. However, high scores on the SDQ have been found to be strongly linked to clinical diagnoses of childhood psychiatric disorders, such as ADHD.^59^ In addition, in 5-year-old children, some of these conditions, such as ADHD, are unlikely to have been diagnosed and following participants from the Cleft Collective at older ages would be required to investigate clinical diagnoses of childhood psychiatric disorders in cleft.

Another limitation linked to the observational nature of the study is missingness. Analyses were performed on children whose mothers returned the baseline questionnaire at 5 years of age and completed the SDQ section. However, other parts of the questionnaire were sometimes missing (eg, cleft type and syndromic status), and this reduced power to detect associations with maternal, familial, and cleft-related factors. In addition, missingness might not have been random; for example, parents might have been less likely to answer the smoking questions if they had smoked during pregnancy. Since this was the first questionnaire that the mothers had to complete (baseline questionnaire), it was not possible to check reasons for non-participation. However, the Cleft Collective has a similar proportion of cleft subtypes and syndromes in the sample to the UK population. Moreover, there was little evidence of a difference in the proportion of cleft subtypes (*P*  =  0.8) or syndromes (*P*  =  0.2) between responders and non-responders to follow-up questionnaires. Despite this limitation, it is reassuring to detect evidence of association of many factors that have been associated with behavioral problems in the general population. We also note that mothers were asked about current smoking and drinking and not about behaviors in pregnancy. However, current smoking and drinking correlate with behaviors in pregnancy.

A final concern is the study's reliance on a single informant (mothers) to provide information on behavioral problems. Many studies delineate the value of using a multi-informant approach to assess a child's behavioral problems, highlighting the improved sensitivity and specificity for detecting disorders that come with this. However, the psychometric properties of the parent version of the SDQ are strong in 5-year-olds^41, 60^ and have been further confirmed in cleft studies.^61^

### Implications for Policy and Practice

Many predictors of behavioral difficulties in the general population, such as higher maternal psychological stress and maternal education, can be extended to the CL/P population. There is a need for targeted interventions, which can provide early support to children born with CL/P who are at an elevated risk of behavioral problems. These results support the need for integrated psychological support (ie, the presence of mental health specialists) on cleft teams and reinforce the imperative for routine psychological assessments of children born with cleft.^62^

## Conclusion

Children born with cleft, particularly boys with CL/P, experience more behavioral problems as indicated by SDQ total difficulties scores and specifically more hyperactivity- and emotional-related difficulties compared to the general population. This study is limited in its ability to make causal inferences about mental health challenges in children born with cleft and leaves room for future inquiry into the direction of the associations observed.^58^ Further investigation is also necessary to clarify the extent and severity of difficulties in high-risk cleft populations and to elucidate the pathways and mechanisms driving outcomes.

## Supplemental Material

sj-docx-1-cpc-10.1177_10556656221119684 - Supplemental material for Prevalence and Factors Associated with Behavioral Problems in 5-Year-Old Children Born with Cleft Lip and/or Palate from the Cleft Collective Supplemental material, sj-docx-1-cpc-10.1177_10556656221119684 for Prevalence and Factors Associated with Behavioral Problems in 5-Year-Old Children Born with Cleft Lip and/or Palate from the Cleft Collective by Samantha Berman, Gemma C. Sharp and Sarah J. Lewis, Rachel Blakey, Amy Davies, Kerry Humphries, Yvonne Wren, Jonathan R. Sandy, Evie Stergiakouli in The Cleft Palate Craniofacial Journal
