# Supplementary material for: Prevalence and Factors Associated with Behavioral Problems in 5-Year-Old Children Born with Cleft Lip and/or Palate from the Cleft Collective
Source: Cleft Palate Craniofac J. 2022 Sep 9;61(1):40–51. doi: 10.1177/10556656221119684 (PMC10676624; doi:10.1177/10556656221119684)
Supplement: sj-docx-1-cpc-10.1177_10556656221119684 - Supplemental material for Prevalence and Factors Associated with Behavioral Problems in 5-Year-Old Children Born with Cleft Lip and/or Palate from the Cleft Collective [file sj-docx-1-cpc-10.1177_10556656221119684.docx]

**Supplement 1: Summary of SDQ Scores and Behavioural Problems in the Cleft Collective 5-year old Cohort Compared to General Population Estimates Stratified by Sex**

| **SDQ subscale-specific thresholds for cases^+^** | **Cohort** | **N Total males** | **N (%) Male cases** | **N (%) Male non-cases** | **N Total Females** | **N (%) Female**  **cases** | **N (%) Female**  **Non-cases** |
| --- | --- | --- | --- | --- | --- | --- | --- |
| **Total difficulties ≥17** | Cleft Collective | 166 | 30 (18.1%) | 136 (81. 9%) | 154 | 16 (10.4%) | 138 (89.6%) |
|  | MCS | 6449 | 610 (9.5%) | 5839 (90.5%) | 6254 | 337 (5.4%) | 5917 (94.6%) |
|  | ONS norms | 5153 | 614 (11.9%) | 4539 (88.3%) | 5145 | 401 (7.8%) | 4745 (91.9%) |
|  | ALSPAC | 4829 | 388 (8%) | 4441 (92.0%) | 4510 | 226 (5.0%) | 4284 (95.0%) |
| **Conduct ≥4** | Cleft Collective | 166 | 25 (15.1%) | 141 (84.9%) | 154 | 14 (9.1%) | 140 (90.9%) |
|  | MCS | 6501 | 789 (12.1%) | 5712 (87.9%) | 6307 | 476 (7.5%) | 5831 (92.5%) |
|  | ONS norms | 5153 | 778 (15.1% | 4375 (84.9%) | 5145 | 531 (10.3%) | 4614 (89.7%) |
|  | ALSPAC | 4849 | 724 (14.9%) | 4123 (85.0%) | 4521 | 520 (11.5%) | 4001 (88.5%) |
| **Emotional ≥5** | Cleft Collective | 166 | 23 (13.9%) | 143 (86.1%) | 154 | 19 (12.3%) | 135 (87.7%) |
|  | MCS | 6488 | 498 (7.7%) | 5990 (92.3%) | 6292 | 452 (7.2%) | 5840 (92.8%) |
|  | ONS norms | 5153 | 548 (10.6%) | 4605 (89.4%) | 5145 | 623 (12.1%) | 4522 (87.9%) |
|  | ALSPAC | 4829 | 223 (4.6%) | 4626 (95.8%) | 4533 | 207 (4.6) | 4326 (95.4%) |
| **Hyperactivity ≥7** | Cleft Collective | 166 | 38 (22.9%) | 128 (77.1%) | 154 | 22 (14.3%) | 132 (85.7%) |
|  | MCS | 6480 | 1069 (16.5%) | 5411 (83.5%) | 6280 | 565 (9.0%) | 5715 (91.0%) |
|  | ONS norms | 5153 | 1005 (19.5%) | 4148 (80.5%) | 5145 | 504 (9.8%) | 4641 (9.1%) |
|  | ALSPAC | 4843 | 828 (17.1) | 4015 (82.9) | 4531 | 514 (11.3%) | 4017 (88.7%) |
| **Peer problems ≥4** | Cleft Collective | 166 | 27 (16.3%) | 139 (83.7%) | 154 | 15 (9.7%) | 139 (90.3%) |
|  | MCS | 6491 | 692 (10.7%) | 5799 (89.3%) | 6295 | 469 (7.5%) | 5826 (92.5%) |
|  | ONS norms | 5153 | 687 (13.3%) | 4466 (86.7%) | 5145 | 520 (10.1%) | 4625 (89.9%) |
|  | ALSPAC | 4850 | 592 (12.2%) | 4258 (87.8%) | 4531 | 420 (9.3%) | 4111 (90.7%) |
| **Prosocial ≤6^*^** | Cleft Collective | 165 | 37 (22.4%) | 128 (77.6%) | 154 | 24 (15.6%) | 130 (84.4) |
|  | MCS | 6504 | 1084 (16.7%) | 5420 (83.3%) | 6307 | 526 (8.3%) | 5781 (91.7%) |
|  | ONS norms | 5153 | 720 (14.0%) | 4433 (86.0%) | 5145 | 364 (7.1%) | 4781 (92.9%) |
|  | ALSPAC | 4846 | 1382 (28.5%) | 3464 (71.5%) | 4523 | 824 (18.2%) | 3699 (81.8%) |

*Abbreviations: SDQ, Strengths and Difficulties Questionnaire; MCS, Millennium Cohort Study; ONS, Office of National Statistics; ALSPAC, Avon Longitudinal Study of Parents and Children.*

*^*^Total difficulties scores were calculated by summing scores from four subscales: conduct problems, hyperactivity, emotional and peer problems. Data on the prosocial subscale were only available on 324 5-year-old children (the prosocial scale is not required to calculate the SDQ total difficulties scores). Lower scores in the prosocial scale (≤6) indicate behavioral problems.*

*^+^Data on SDQ and sex were available for 320 children.*

**Supplement 2: Odds Ratios for Behavioural Problems in the Cleft Collective Compared to General Population Samples Stratified by Sex**

| **SDQ subscale-specific thresholds for cases** | **Cohort** | **Male Only** | | | **Female Only** | | |
| --- | --- | --- | --- | --- | --- | --- | --- |
|  |  | **Odds Ratio** | **95% CIs** | **p value** | **Odds Ratio** | **95% CIs** | **p value** |
| **Total difficulties ≥17** | Cleft Collective | 1.00 |  |  | 1.00 |  |  |
|  | MCS | 2.11 | 1.41 3.16 | <0.001 | 2.04 | 1.20, 3.46 | 0.008 |
|  | ONS norms | 1.63 | 1.09 2.44 | 0.018 | 1.37 | 0.81, 2.33 | 0.592 |
|  | ALSPAC | 2.52 | 1.67 3.80 | <0.001 | 2.20 | 1.29, 3.75 | <0.001 |
| **Conduct ≥4** | Cleft Collective | 1 |  |  | 1.00 |  |  |
|  | MCS | 1.28 | 0.82 1.98 | 0.299 | 1.23 | 0.70, 2.14 | 0.475 |
|  | ONS norms | 1.00 | 0.65 1.54 | 0.913 | 0.87 | 0.50, 1.52 | 0.621 |
|  | ALSPAC | 1.01 | 0.66 1.56 | 0.959 | 0.77 | 0.44, 1.34 | 0.357 |
| **Emotional ≥5** | Cleft Collective | 1.00 |  |  | 1.00 |  |  |
|  | MCS | 1.93 | 1.23 3.03 | 0.004 | 1.39 | 0.81, 2.40 | 0.229 |
|  | ONS norms | 1.35 | 0.86 2.12 | 0.199 | 0.78 | 0.46, 1.34 | 0.375 |
|  | ALSPAC | 3.34 | 2.11 5.29 | <0.001 | 2.26 | 1.30, 3.91 | 0.004 |
| **Hyperactivity ≥7** | Cleft Collective | 1.00 |  |  | 1.00 |  |  |
|  | MCS | 1.50 | 1.04 2.17 | 0.022 | 1.69 | 1.06, 2.67 | 0.026 |
|  | ONS norms | 1.23 | 0.85 1.77 | 0.234 | 1.53 | 0.97, 2.43 | 0.068 |
|  | ALSPAC | 1.44 | 0.99 2.08 | 0.051 | 1.30 | 0.82, 2.06 | 0.261 |
| **Peer problems ≥4** | Cleft Collective | 1.00 |  |  | 1.00 |  |  |
|  | MCS | 1.63 | 1.07 2.48 | 0.016 | 1.34 | 0.78, 2.30 | 0.288 |
|  | ONS norms | 1.26 | 0.83 1.92 | 0.226 | 0.96 | 0.56, 1.65 | 0.882 |
|  | ALSPAC | 1.40 | 0.92 2.13 | 0.092 | 1.06 | 0.61, 1.82 | 0.843 |
| **Prosocial ≤6^*^** | Cleft Collective | 1.00 |  |  | 1.00 |  |  |
|  | MCS | 1.45 | 1.00 2.10 | 0.050 | 2.03 | 1.30, 3.16 | <0.001 |
|  | ONS norms | 1.78 | 1.22 2.59 | <0.001 | 2.42 | 1.55, 3.80 | <0.001 |
|  | ALSPAC | 0.72 | 0.50 1.05 | 0.145 | 0.83 | 0.53, 1.29 | 0.358 |

*Abbreviations: SDQ, Strengths and Difficulties Questionnaire; Cis, Confidence Intervals; MCS, Millennium Cohort Study; ONS, Office of National Statistics; ALSPAC, Avon Longitudinal Study of Parents and Children.*

*^*^Total difficulties scores were calculated by summing scores from four subscales: conduct problems, hyperactivity, emotional and peer problems. Data on the prosocial subscale were only available on 324 5-year-old children (the prosocial scale is not required to calculate the SDQ total difficulties scores). Lower scores in the prosocial scale (≤6) indicate behavioral problems.*

**Supplement 3: Logistic regression results to assess associations between dichotomized SDQ Total Difficulties scores (dependent variable) and maternal, familial and cleft-related (independent) variables. In each analysis, binary SDQ scores defined the dependent variable and the maternal factors were used as binary independent variables, except cleft-type which was treated as a nominal categorical variable. Odds ratios (OR) with 95% confidence intervals and p values for each of the variable values indicated in the first column. Odds Ratios, p-values, and 95% confidence intervals for associations between Sociodemographic and Maternal Characteristics and Behavioral problems among CL/P 5-Year-Olds**

| **Variables (baseline value, other value)** | **Odds Ratio** | **95% CIs** | **p-values** |
| --- | --- | --- | --- |
| Sex (Male, Female) | 0.53 | 0.27 1.01 | 0.053 |
| Cleft Type (Cleft Lip) | 1.00 |  |  |
| Cleft Type (Cleft Lip, Cleft Palate) | 0.94 | 0.30 2.97 | 0.922 |
| Cleft Type (Cleft Lip, Cleft Lip and Palate) | 1.11 | 0.38 3.28 | 0.846 |
| Syndrome (No, Yes) | 3.00 | 0.67 13.53 | 0.153 |
| Maternal Age at conception (>24 years, ≤24 years) | 3.73 | 1.81 7.72 | <0.001 |
| Maternal Ethnicity (White, Black, Asian, or Minority Ethnic) | 0.94 | 0.27 3.32 | 0.923 |
| Maternal Education (No University Degree, University Degree or equivalent) | 2.34 | 1.17 4.69 | 0.016 |
| Household Income (<£20,000 annually, ≥£20,000 annually) | 1.15 | 0.57 2.34 | 0.696 |
| Mother Receives Income Support (No, Yes) | 6.35 | 2.42 16.68 | <0.001 |
| Parity (0, ≥1) | 0.65 | 0.34 1.27 | 0.206 |
| Mother Smokes (No, Yes) | 4.41 | 1.75 11.13 | 0.002 |
| Marital Status (Married/Partnered, Single/Divorced/Widowed) | 3.19 | 1.51 6.72 | 0.002 |
| Mother consumes alcohol (No, Yes) | 1.03 | 0.52 2.02 | 0.942 |
| Maternal PSS (<50%,>=50%) | 10.13 | 3.87 26.51 | <0.001 |
| Total PedsQL FIM (<50%,>=50%) | 5.20 | 2.42 11.19 | <0.001 |
| PedsQL (Family Functioning) (<50%,>=50%) | 4.58 | 2.31 9.12 | <0.001 |
| PedsQL (Maternal HRQoL) (<50%,>=50%) | 7.48 | 3.03 18.49 | <0.001 |

*Abbreviations: SDQ, Strengths and Difficulties Questionnaire; PSS,* *Perceived Stress Scale; PedsQL FIM,* *Pediatric Quality of Life Inventory Family Impact Module; HRQoL, Health-Related Quality Of Life*

**Supplement 4: Logistic regression results to assess associations between dichotomized SDQ subscale scores (dependent variable) and maternal, familial and cleft-related (independent) variables. In each analysis, binary SDQ scores defined the dependent variable and the maternal factors were used as binary independent variables, except cleft-type which was treated as a nominal categorical variable. Odds ratios (OR) with 95% confidence intervals and p values for each of the variable values indicated in the first column. Odds Ratios, p-values, and 95% confidence intervals for associations between Sociodemographic and Maternal Characteristics and Behavioral problems among CL/P 5-Year-Olds**

| **Emotional ≥5** | **Odds Ratio** | **95% CIs** | **p-values** |
| --- | --- | --- | --- |
| Sex (Male, Female) | 0.88 | 0.46 1.68 | 0.688 |
| Cleft Type (Cleft Lip) | 1.00 |  |  |
| Cleft Type (Cleft Lip, Cleft Palate) | 0.81 | 0.25 2.62 | 0.724 |
| Cleft Type (Cleft Lip, Cleft Lip and Palate) | 0.99 | 0.33 2.98 | 0.992 |
| Syndrome (No, Yes) | 0.88 | 0.10 7.75 | 0.906 |
| Maternal Age at conception (>24 years, ≤24 years) | 5.88 | 2.86 12.09 | <0.001 |
| Maternal Ethnicity (White, Black, Asian, or Minority Ethnic) | 1.49 | 0.48 4.62 | 0.495 |
| Maternal Education (No University Degree, University Degree or equivalent) | 2.47 | 1.18 5.19 | 0.017 |
| Household Income (<£20,000 annually, ≥£20,000 annually) | 1.05 | 0.51 2.17 | 0.898 |
| Mother Receives Income Support (No, Yes) | 6.78 | 2.57 17.86 | <0.001 |
| Parity (0, ≥1) | 0.68 | 0.35 1.33 | 0.261 |
| Mother Smokes (No, Yes) | 3.48 | 1.35 9.00 | 0.010 |
| Marital Status (Married/Partnered, Single/Divorced/Widowed) | 2.23 | 1.01 4.94 | 0.048 |
| Mother consumes alcohol (No, Yes) | 0.72 | 0.37 1.40 | 0.332 |
| Maternal PSS (<50%,>=50%) | 2.75 | 1.34 5.62 | 0.006 |
| Total PedsQL FIM (<50%,>=50%) | 5.48 | 2.45 12.24 | <0.001 |
| PedsQL (Family Functioning) (<50%,>=50%) | 3.57 | 1.81 7.08 | <0.001 |
| PedsQL (Maternal HRQoL) (<50%,>=50%) | 5.64 | 2.39 13.31 | <0.001 |
| **Conduct ≥4** | **Odds Ratio** | **95% CIs** | **p-values** |
| Sex (Male, Female) | 0.56 | 0.28 1.13 | 0.106 |
| Cleft Type (Cleft Lip) | 1.00 |  |  |
| Cleft Type (Cleft Lip, Cleft Palate) | 0.95 | 0.20 4.49 | 0.947 |
| Cleft Type (Cleft Lip, Cleft Lip and Palate) | 2.16 | 0.56 8.39 | 0.264 |
| Syndrome (No, Yes) | 2.61 | 0.46 14.75 | 0.278 |
| Maternal Age at conception (>24 years, ≤24 years) | 2.01 | 0.87 4.60 | 0.100 |
| Maternal Ethnicity (White, Black, Asian, or Minority Ethnic) | 0.71 | 0.16 3.17 | 0.655 |
| Maternal Education (No University Degree, University Degree or equivalent) | 2.74 | 1.28 5.88 | 0.010 |
| Household Income (<£20,000 annually, ≥£20,000 annually) | 1.30 | 0.60 2.81 | 0.509 |
| Mother Receives Income Support (No, Yes) | 8.10 | 3.04 21.56 | <0.001 |
| Parity (0, ≥1) | 1.34 | 0.68 2.65 | 0.396 |
| Mother Smokes (No, Yes) | 7.26 | 2.66 19.78 | <0.001 |
| Marital Status (Married/Partnered, Single/Divorced/Widowed) | 2.68 | 1.20 6.02 | 0.017 |
| Mother consumes alcohol (No, Yes) | 0.91 | 0.45 1.86 | 0.802 |
| Maternal PSS (<50%,>=50%) | 5.09 | 2.16 11.99 | <0.001 |
| Total PedsQL FIM (<50%,>=50%) | 3.82 | 1.75 8.37 | 0.001 |
| PedsQL (Family Functioning) (<50%,>=50%) | 4.22 | 2.01 8.87 | <0.001 |
| PedsQL (Maternal HRQoL) (<50%,>=50%) | 4.30 | 1.79 10.32 | 0.001 |
| **Hyperactivity ≥7** | **Odds Ratio** | **95% CIs** | **p-values** |
| Sex (Male, Female) | 0.56 | 0.31 1.00 | 0.051 |
| Cleft Type (Cleft Lip) | 1.00 |  |  |
| Cleft Type (Cleft Lip, Cleft Palate) | 2.33 | 0.76 7.12 | 0.136 |
| Cleft Type (Cleft Lip, Cleft Lip and Palate) | 1.69 | 0.55 5.14 | 0.359 |
| Syndrome (No, Yes) | 2.47 | 0.63 9.76 | 0.197 |
| Maternal Age at conception (>24 years, ≤24 years) | 1.34 | 0.64 2.82 | 0.435 |
| Maternal Ethnicity (White, Black, Asian, or Minority Ethnic) | 1.30 | 0.46 3.67 | 0.625 |
| Maternal Education (No University Degree, University Degree or equivalent) | 1.99 | 1.09 3.66 | 0.026 |
| Household Income (<£20,000 annually, ≥£20,000 annually) | 0.93 | 0.48 1.79 | 0.833 |
| Mother Receives Income Support (No, Yes) | 2.80 | 1.05 7.47 | 0.040 |
| Parity (0, ≥1) | 1.16 | 0.65 2.05 | 0.622 |
| Mother Smokes (No, Yes) | 3.29 | 1.37 7.90 | 0.008 |
| Marital Status (Married/Partnered, Single/Divorced/Widowed) | 1.40 | 0.65 3.04 | 0.389 |
| Mother consumes alcohol (No, Yes) | 0.69 | 0.39 1.24 | 0.214 |
| Maternal PSS (<50%,>=50%) | 3.03 | 1.61 5.70 | 0.001 |
| Total PedsQL FIM (<50%,>=50%) | 3.09 | 1.67 5.71 | <0.001 |
| PedsQL (Family Functioning) (<50%,>=50%) | 2.75 | 1.54 4.92 | 0.001 |
| PedsQL (Maternal HRQoL) (<50%,>=50%) | 3.86 | 1.91 7.80 | <0.001 |
| **Peer Problems ≥4** | **Odds Ratio** | **95% CIs** | **p-values** |
| Sex (Male, Female) | 0.56 | 0.28 1.09 | 0.087 |
| Cleft Type (Cleft Lip) | 1.00 |  |  |
| Cleft Type (Cleft Lip, Cleft Palate) | 1.48 | 0.35 6.31 | 0.596 |
| Cleft Type (Cleft Lip, Cleft Lip and Palate) | 2.16 | 0.56 8.39 | 0.264 |
| Syndrome (No, Yes) | 3.86 | 0.83 17.94 | 0.085 |
| Maternal Age at conception (>24 years, ≤24 years) | 2.64 | 1.24 5.65 | 0.012 |
| Maternal Ethnicity (White, Black, Asian, or Minority Ethnic) | 0.65 | 0.15 2.88 | 0.570 |
| Maternal Education (No University Degree, University Degree or equivalent) | 1.73 | 0.86 3.48 | 0.122 |
| Household Income (<£20,000 annually, ≥£20,000 annually) | 0.73 | 0.33 1.60 | 0.428 |
| Mother Receives Income Support (No, Yes) | 2.44 | 0.83 7.18 | 0.104 |
| Parity (0, ≥1) | 0.54 | 0.27 1.10 | 0.089 |
| Mother Smokes (No, Yes) | 2.67 | 1.00 7.16 | 0.051 |
| Marital Status (Married/Partnered, Single/Divorced/Widowed) | 0.64 | 0.22 1.90 | 0.424 |
| Mother consumes alcohol (No, Yes) | 1.25 | 0.60 2.61 | 0.554 |
| Maternal PSS (<50%,>=50%) | 5.74 | 2.45 13.43 | <0.001 |
| Total PedsQL FIM (<50%,>=50%) | 3.86 | 1.83 8.16 | <0.001 |
| PedsQL (Family Functioning) (<50%,>=50%) | 5.82 | 2.74 12.33 | <0.001 |
| PedsQL (Maternal HRQoL) (<50%,>=50%) | 7.68 | 2.88 20.47 | <0.001 |
| **Prosocial ≤6** | **Odds Ratio** | **95% CIs** | **p-values** |
| Sex (Male, Female) | 0.64 | 0.36 1.13 | 0.122 |
| Cleft Type (Cleft Lip) | 1.00 |  |  |
| Cleft Type (Cleft Lip, Cleft Palate) | 1.15 | 0.40 3.29 | 0.791 |
| Cleft Type (Cleft Lip, Cleft Lip and Palate) | 1.25 | 0.46 3.41 | 0.669 |
| Syndrome (No, Yes) | 1.00 | 0.19 5.17 | 1.000 |
| Maternal Age at conception (>24 years, ≤24 years) | 2.49 | 1.26 4.92 | 0.009 |
| Maternal Ethnicity (White, Black, Asian, or Minority Ethnic) | 2.57 | 1.02 6.43 | 0.044 |
| Maternal Education (No University Degree, University Degree or equivalent) | 1.55 | 0.86 2.80 | 0.143 |
| Household Income (<£20,000 annually, ≥£20,000 annually) | 1.02 | 0.54 1.91 | 0.956 |
| Mother Receives Income Support (No, Yes) | 3.33 | 1.28 8.68 | 0.014 |
| Parity (0, ≥1) | 1.31 | 0.75 2.31 | 0.344 |
| Mother Smokes (No, Yes) | 1.84 | 0.81 4.14 | 0.144 |
| Marital Status (Married/Partnered, Single/Divorced/Widowed) | 1.79 | 0.86 3.72 | 0.122 |
| Mother consumes alcohol (No, Yes) | 0.43 | 0.24 0.76 | 0.004 |
| Maternal PSS (<50%,>=50%) | 2.55 | 1.38 4.70 | 0.003 |
| Total PedsQL FIM (<50%,>=50%) | 2.09 | 1.17 3.71 | 0.013 |
| PedsQL (Family Functioning) (<50%,>=50%) | 2.50 | 1.42 4.42 | 0.002 |
| PedsQL (Maternal HRQoL) (<50%,>=50%) | 2.62 | 1.39 4.94 | 0.003 |

*Abbreviations: SDQ, Strengths and Difficulties Questionnaire; PSS,* *Perceived Stress Scale; PedsQL FIM,* *Pediatric Quality of Life Inventory Family Impact Module; HRQoL, Health-Related Quality Of Life*
